# In Vitro Assessment of Cisplatin/Hyaluronan Complex for Loco-Regional Chemotherapy

**DOI:** 10.3390/ijms242115725

**Published:** 2023-10-29

**Authors:** Sabrina Banella, Aishwarya Saraswat, Akanksha Patel, Abu T. M. Serajuddin, Paolo Colombo, Ketan Patel, Gaia Colombo

**Affiliations:** 1 Department of Life Sciences and Biotechnology, University of Ferrara, 44121 Ferrara, Italy; bnlsrn@unife.it (S.B.); clmgai@unife.it (G.C.); 2 College of Pharmacy and Health Sciences, St. John’s University, Queens, NY 11439, USA; aishwarya.saraswat19@my.stjohns.edu (A.S.); akanksha.patel21@my.stjohns.edu (A.P.); serajuda@stjohns.edu (A.T.M.S.); 3 PlumeStars s.r.l., 43125 Parma, Italy; paolo.colombo@unipr.it

**Keywords:** cisplatin, hyaluronan, coordination complex, loco-regional therapy, chemotherapy, CD44 receptor, drug delivery system, melanoma, pleural mesothelioma

## Abstract

Loco-regional chemotherapy is a strategy used to achieve more precise anticancer drug effect directly on tumor mass, while decreasing whole body exposure, which can lead to undesirable side effects. Thus, the loco-regional chemotherapy is conceptually similar to the targeted drug delivery systems for delivering chemotherapeutics to cancer cells in a certain location of the body. Recently, it has been demonstrated that a novel polymeric film containing the complex between cisplatin (cisPt) and hyaluronan (sodium salt of hyaluronic acid; NaHA) enhanced in vivo efficacy and safety of cisplatin (cisPt) by loco-regional delivery in pleural mesothelioma. Biologically, hyaluronic acid (HA) binds with the CD44 receptor, which is a transmembrane glycoprotein overexpressed by other cancer cells. Thus, administering both cisPt and hyaluronan together as a complex loco-regionally to the tumor site could target cancer cells locally and enhance treatment safety. A slight excess of hyaluronan was required to have more than 85% cisPt complexation. In cell monolayers (2D model) the cisPt/NaHA complex in solution demonstrated dose- and time-dependent cytotoxic effect by decreasing the viability of pancreatic, melanoma, and lung cell lines (they all express CD44). At the same concentration in solution, the complex was as effective as cisPt alone. However, when applied as film to melanoma spheroids (3D model), the complex was superior because it prevented the tumor spheroid growth and, more importantly, the formation of new cell colonies. Hence, cisPt/NaHA complex could work in preventing metastases loco-regionally and potentially avoiding systemic relapses.

## 1. Introduction

Cancer is one of the major causes of death, with an estimated 28.9 million new cancer cases projected by 2040 [1]. The available conventional chemotherapeutic drugs often lack specificity for the tumor, thus exposing the body to heavy side effects. Moreover, tumors often relapse. Lack of specificity and regional and/or systemic recurrences can compromise the success of cancer therapy, as is the case of pleural mesothelioma (PM). Cisplatin (cisPt) is a first-line drug for PM, but it is also highly toxic and may cause nephrotoxicity, ototoxicity, severe nausea and vomiting, and myelosuppression [2]. Its severe side effects are dose-limiting to the point of being discontinued and substituted with other platinum-derivatives or alternative anticancer medicines to complete the therapy. Additionally, regional relapses, where the tumor grows in tissues near the primary cancer site, develop frequently from PM.

To increase the efficacy of anticancer therapy, multimodal treatment and/or innovative strategies such as nanotechnological delivery systems, targeted therapy, and immunotherapy have been developed [3,4,5,6]. In the multimodal approach, the chemotherapy, which remains the blockbuster for all tumors, is combined with surgery, whenever possible, and radiotherapy. The main goal of such an approach is to remove the tumor surgically and prevent relapses by chemo/radiotherapy. For example, surgery and loco-regional chemotherapy are combined in PM management such that, after tumor resection, the pleural cavity is perfused with a cisplatin solution to remove residual malignant cells. However, the procedure per se represents a hazard for the medical team in the operating room, since the solution can leak during the perfusion of thoracic cavity, leading to possible contamination of surfaces and exposure of the surgical team to the antineoplastic drug [7]. Additionally, cisPt as a liquid solution is easily cleared from the application site, thus rapidly decreasing its local effect and exposing other parts of the body to the drug.

The concept of a loco-regional and targeted therapy of cancer is reasonable and worth the search for suitable delivery technologies to accomplish it. There is not a generally accepted definition of loco-regional therapy, but it usually refers to all therapies that are minimally invasive. By localizing the therapy in the region of the target cells, an effective concentration is achieved at the site of action and the total dose is reduced. Additionally, by controlling drug release on site, safety can be enhanced by preventing body exposure to toxic drug levels. To this aim, the cisPt-loaded polymeric film made of sodium hyaluronate (NaHA) was invented and found suitable for the loco-regional treatment of PM. The film can be applied after surgical resection of PM onto the mesothelial surfaces, releasing cisPt locally over an extended period. Preclinical data in the rat PM model proved the effectiveness of the film in reducing tumor volume and relapses as compared to intrapleural and intravenous administration of cisplatin solution [8]. More importantly, although cisPt released from the film led to high levels in plasma, there was no organ toxicity (kidneys, liver, and heart) compared to administering the cisPt solution alone [9]. This result was achieved because of the interaction of cisplatin with hyaluronan (cisPt/NaHA complex), as we have demonstrated in vitro [10]. The complex exists in the film as well as in solution as the film dissolves, and the film also controls cisPt release, influencing its bioavailability.

An additional value of a hyaluronan-based delivery system in cancer therapy is that hyaluronic acid is the main ligand of CD44 receptor. Thus, the affinity for CD44 adds favorable targeting properties to the cisPt/NaHA complex [11]. In other words, the complex within the delivery system enables a loco-regional therapy with cell-recognition capacity. Sakurai and co-workers demonstrated that cisPt-loaded lipid nanoparticles incorporating hyaluronic acid promoted cellular uptake of the drug [12]. They, however, exploited the polysaccharide for its targeting properties only and not for its capability to complex or coordinate with cisPt.

The aim of the present study was to investigate in vitro the cytotoxicity of the cisPt/NaHA complex. To do so, the complex in solution was applied onto 2D cell models of various tumors, namely pancreatic (BxPC_3_ and MIA PaCa-2), lung (A549), and melanoma (A375) cells. cisPt alone as an aqueous solution was the reference treatment. Additionally, the combination of valproic acid to cisPt was included in the investigated treatments for the reported evidence of synergism [13,14]. The effect of polymer molecular weight was explored in the study as a formulation variable by comparing a high molecular weight (HMW) hyaluronan with a low molecular weight (LMW) one. After identifying the cell line on which the activity was the greatest, 3D models (spheroids) were grown with these cells. Differently from cell monolayers, spheroids could be used for drug diffusion/penetration through the “tumor mass”. The molecular mechanisms of action of cisplatin are described in the literature [15]. Therefore, no investigation on the mechanistic effect cisPt effect has been conducted in the present investigation.

## 2. Results and Discussion

According to our previous findings, the cisplatin/hyaluronan complex exists in solution (i.e., simple drug + polymer solutions and viscous film-forming mixture) as well as in solid state (i.e., thin film) [10]. While the thin film is a suitable form for the application on relatively large surfaces like in an exposed cavity, liquid forms are more favorable to deliver cisplatin and the complex by injection with a syringe or via cannula and catheter (e.g., to reach the bladder via the urinary tract). Melanoma and bladder cancer are tumors where the cisPt/NaHA complex could be exploited for a loco-regional therapy via application in a simple, effective form like a viscous solution. For this purpose, it was necessary to assess whether the complex was superior to the free drug in solution against pancreatic, lung, and melanoma cells.

### 2.1. Effect of Monomer:Drug Molar Ratio and Polymer Molecular Weight in the Complexation

Prior to the current study, the cisPt/NaHA complex was assessed in the film-forming mixture (FFM), which contained several excipients in addition to drug and hyaluronan. The molar ratio was fixed by the film composition. In the present investigation, it was studied if the molar ratio could be varied without affecting the complex formation. Additionally, it was of interest to determine whether the complexation could be influenced by the polymer molecular weight (MW). The hyaluronic acid MW was considered as a variable since it could affect the interaction with CD44 receptor [16].

**Figure 1:**
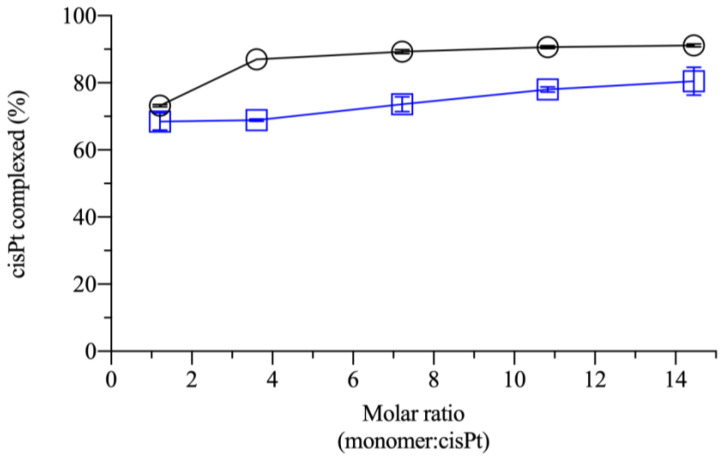
Fraction of cisPt coordinated by NaHA as a function of (monomer:drug) molar ratio for: high (HMW; black) and low molecular weight (LMW; blue).

The efficiency of cisPt complexation by NaHA, such as how much hyaluronan was needed to fully complex the available drug, was first conducted for NaHA of different molecular weights. The information would also be helpful to compare our data with those of other researchers, who used a low 35 kDa MW NaHA [17,18]. In this experiment, cisPt concentration was kept fixed and the NaHA concentration was varied to determine the effects of interaction between different molar ratios (monomer:drug) of the two species (from 1.2:1 to 14.5:1, where 1 refers to the fixed drug part). To keep the viscosity of the formulations under control, NaHA concentrations for both molecular weights (i.e., 10 kDa and 1330 kDa) did not exceed half of the NaHA concentration in FFM. In the FFM, the NaHA/cisPt molar ratio was 30:1 (monomer:drug). The mixing time was kept at 18 h, according to the procedure of FFM preparation. Figure 1 indicates that the minimum amount of HMW polymer required to complex at least 85% of available cisPt was 3.6 parts of polymer for 1 part of the drug.

A further increase of the polymer concentration with respect to the drug increased the complexation marginally. For the LMW NaHA, the 3.6:1 molar ratio was insufficient to have the same % cisPt complexed as compared to the HMW polymer. CisPt complexation by hyaluronan for 18 h depended on the polymer molecular weight. This is because, assuming that one cisPt molecule is coordinated by two carboxylate groups, i.e., two monomers, the stoichiometry of complexation would correspond to 2:1 molar ratio (monomer:drug). The data in Figure 1 showed that complexation was not total at the ratios around the stoichiometric one (1.2:1 and 3.6:1), particularly for LMW NaHA. It is possible that, despite the 2:1 theoretical ratio, not all carboxylates were equally accessible to bind platinum. Steric effects could hinder interaction with part of the groups and steric hindrance may be different between the LMW and HMW polymers. Consequently, improved complexation efficiency was seen at ratios where hyaluronan was well in excess compared to cisPt. As a last point, calculating the molar ratio with respect to the monomer neglected the fact that the number of monomers increased with the polymer chain length. This can explain why HMW was more efficient than LMW in complexing with cisPt at low molar ratios (monomer:drug). As the ratio increased, the MW-dependence should disappear. The molar ratio (monomer:drug) in the film-forming mixture was 30:1, i.e., a huge excess of the HMW polymer as compared to drug, where the complexation capacity was expected to be maximum.

### 2.2. In Vitro Cytotoxicity of the cisPt/NaHA Complex

The activity of the cisPt/NaHA complex against cancer cells was tested using the cisPt/NaHA solution at 3.6:1 ratio (monomer:drug). This mixture was deemed more suitable for the study than the film-forming mixture that contained other excipients [10]. Cell lines were chosen because they highly expressed the CD44 transmembrane receptor [19,20,21]. CD44 is a cell surface glycoprotein overexpressed in many solid tumors including pancreatic, breast, ovarian, brain, and lung cancers. It is a multi-structural glycoprotein of the cell surface, which is majorly involved in cell proliferation, cell-to-cell interaction, cellular migration, inflammation, and generation of immune responses [16]. The CD44 is the main receptor for hyaluronic acid in healthy tissues and their binding activates and potentiates cell proliferation. This is why it is overexpressed by many tumors and considered a target for anticancer therapy by researchers [22]. Hyaluronan-based drug delivery systems have the advantage over other carrier polymers of the high affinity for the CD44 receptor. They have a potentially greater chance of delivering chemotherapeutic agents directly to the cell in a targeted manner by sparing normal cells from their adverse effects [23]. Additionally, hyaluronan is biocompatible and biodegradable.

In the first in vitro experiment, 2D cell cultures were treated with six increasing concentrations of the following:

- cisplatin alone;
- cisPt complexed with high molecular weight hyaluronan;
- cisPt combined with valproic acid (VA).

The cytotoxic effect was expressed in terms of cell viability in comparison with untreated cells. For this experiment (and for the next one on the 2D models), only cisPt was combined with VA—not the cisPt/NaHA complex.

**Figure 2:**
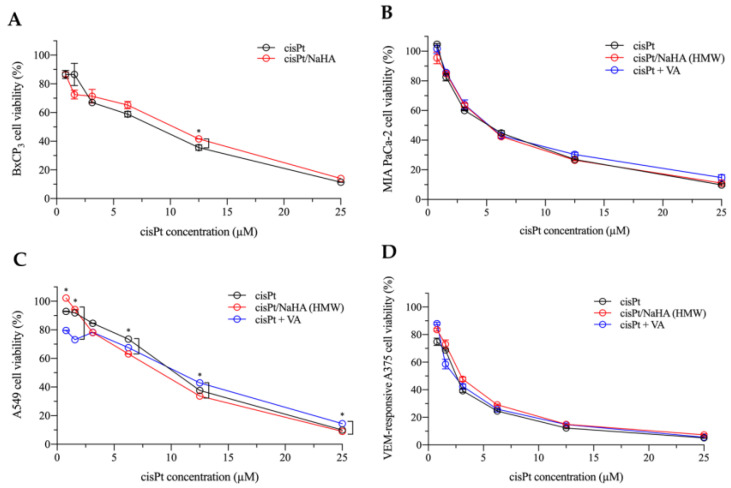
Concentration-dependent cytotoxicity of cisPt alone (black), cisPt/NaHA HMW (red), and cisPt + VA (blue) in: (**A**) BxPC_3_, (**B**) MIA PaCa-2, (**C**) A549, and (**D**) VEM-responsive A375 cell lines. Cell viability was determined by MTT assay after 48 h exposure to treatment. Data are reported as mean ± SEM (*n* = 6). Asterisks indicate a statistically significant difference (*p* < 0.05).

In all cell lines, the cytotoxicity of the treatments increased with cisPt concentration (Figure 2). The greatest cytotoxic effect was observed in MIA PaCa-2 and VEM-responsive A375 cells as compared to BxPC_3_ and A549 cell lines (*p* < 0.05). VEM-responsive A375 cells were the most sensitive cell line among all the treatments.

Independently of cisPt concentration, % of cell death was comparable in cisPt and cisPt/NaHA groups, with no statistically significant difference. Overall, the complex was not more active than the drug alone, although in the less sensitive cell lines to cisPt, i.e., pancreatic BxPC_3_ cells, the cisPt/NaHA complex was less cytotoxic than the cisPt alone at 12.5 µM (*p* < 0.05). Instead, in adenocarcinoma lung A549 cells, when the concentration of cisplatin was 6.25 µM, the cisPt/NaHA complex was more cytotoxic than cisplatin alone (*p* < 0.05). The expected synergistic effect between cisplatin and VA in killing cells was not observed in any of the cell lines, indicating that the cell viability was not significantly different from the treatment with cisPt alone (*p* > 0.05). Consequently, no synergy score could be calculated. Indeed, an in vitro study by Wawruszak et al. showed that the combination between cisplatin and valproic acid can lead to an additive, synergistic, or even antagonistic effect depending on the cell line used [24].

From the cell viability data, the corresponding IC_50_ for each cell line was calculated and is reported in Table 1.

As shown in Table 1, in these 2D models both cisplatin alone and cisPt complexed with hyaluronan showed comparable cytotoxicity, and the presence of VA made no difference. A similar observation was also made by Ishiguro and co-workers in four lung cancer cell lines, i.e., LLC, H1299, H358, and A549. In their work, they used a hyaluronic acid with low molecular weight (35 kDa) [25].

In our attempt to understand the above result, we raised the following points:

1. Was cisPt really complexed by hyaluronan in the three stock solutions as it was in the film-forming mixture and film applied in vivo?
2. Did the dilution of the stock solution with the cell culture medium dissociate any complex formed?
3. Did the polymer high molecular weight of hyaluronan affect cisPt availability at intracellular level, also considering the CD44 receptor presence?

Regarding the first question, the RP-HPLC analysis of cisPt/NaHA stock solutions confirmed that cisPt was complexed, with about 24% of free drug detected in the solution. This result was also in accordance with the previous experiment on cisPt/NaHA complexation (see Section 2.1). Hence, at time 0, 24% free cisPt was possibly available immediately to kill cells. The rest would be released from the macromolecular complex over the experiment course. However, these solutions were diluted 44 folds with cell culture medium to test them on cells.

Dilution of the stock solution containing the cisPt/NaHA complex (the second question) reduced the concentration of both hyaluronan and drug, but did not change their molar ratio. The culture medium used for dilution contained chloride ions and albumin. Chloride ions displace platinum from the complex, while serum components bind cisPt. Both factors could dissociate the complex. Unfortunately, due to analytical interferences by the cell culture medium, the quantification of free cisPt in the diluted solutions was not possible by RP-HPLC to verify any complex dissociation upon dilution. If the dissociation of complex had occurred, the two treatments would have been equally effective.

**Figure 3:**
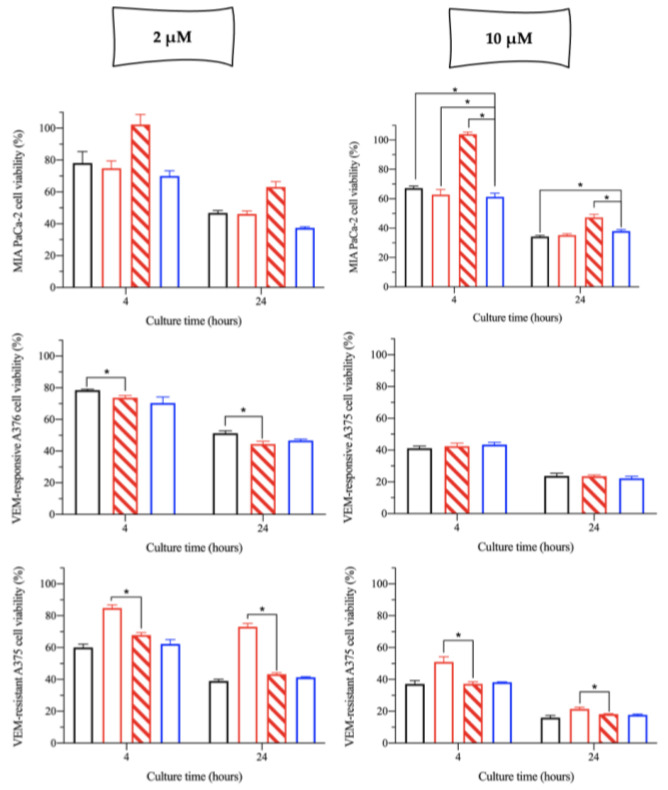
Cytotoxic effect of cisPt alone (black bar), cisPt/NaHA HMW (empty red bar) and cisPt/NaHA LMW (filled red bar), and cisPt + VA (blue bar) in MIA PaCa-2 (top plots), VEM-responsive A375 (middle plots), and VEM-resistant A375 (bottom plots). Cell viability was determined by MTT assay after 4 and 24 h exposure to 2 µM (left plots) and 10 µM (right plots) cisPt. Data are reported as mean ± SEM (*n* = 6). Asterisks indicate a statistically significant difference (*p* < 0.05).

Finally, the third question concerns whether the NaHA molecular weight was a variable “hiding” the cytotoxicity of the complex. In the second experiment, cells were treated with a cisPt/NaHA solution prepared with a much lower molecular weight NaHA (10 kDa). Only two cisPt concentrations were tested, namely 2 and 10 µM. The treatment duration was shortened from 48 h to 24 h to assess any effect of the exposure time (Figure 3). BxPC_3_ and A549 cells were not considered for this experiment due to the high IC_50_ observed (Table 1).

As shown in Figure 3, the cytotoxic effect of all treatments (i.e., free cisPt, cisPt/NaHA (HMW and/or LMW, depending on cell line) complex and cisPt + VA) was time dependent, with more cells killed at 24 h than at 4 h. Merging data from this and the previous experiment (Figure 2), for cisPt alone and cisPt + VA, the viability of cells at 24 h was higher than that at 48 h, confirming the effect of time on cytotoxicity.

In the pancreatic cancer cell line MIA PaCa-2, there was an effect of the NaHA polymer molecular weight. At both cisPt concentrations, 100% of cells treated with the LMW NaHA complex were viable after 4 h of exposure, indicating that there was no effect of the complex in the first 4 h. Prolonging the treatment to 24 h brought the toxicity of this complex to values that were more in line with the other treatments, but still slightly less cytotoxic. Thus, with these cells the complex with HMW NaHA would be more preferable.

On VEM-responsive A375 cells only LMW NaHA was tested, thus a direct comparison with the HMW was not possible. Nevertheless, HMW hyaluronan had been used in the previous experiment (Figure 2) and the cytotoxicity of 2.5 µM cisPt/NaHA HMW after 48 h was in line with the one achieved here after 24 h exposure to 2 µM cisPt/NaHA LMW complex. The same can be said for the effect of 10 µM cisPt/NaHA complex. Hence, it can be argued that in VEM-responsive A375 melanoma cell line, the two hyaluronan molecular weights were equivalent in terms of cytotoxicity of the complex.

Finally, in VEM-resistant A375 cells, the LMW hyaluronan complexed with cisPt was more cytotoxic than the HMW at both cisPt concentrations and both exposure times.

Taken together, these data appear diverse. The cell lines confirmed a different sensitivity to the treatments, which makes it difficult to draw a conclusion on the effect of hyaluronan molecular weight. In fact, on MIA PaCa-2 cells, the activity was higher for the cisPt/NaHA complex made with the HMW polymer (*p* < 0.05), whereas VEM-responsive A375 cells gave the opposite result (more activity with LMW NaHA; *p* < 0.05). Eventually, cisPt alone resulted equally cytotoxic in all cases.

In summary, the lack of difference between activities of cisPt and the complex must be ascribed to other reasons, or this may be the only possible result, at least in vitro. In the in vivo experiments with the film, its superior anticancer activity and safety compared to cisPt alone had been attributed to a different pharmacokinetics of the drug released as complex [8,9]. In contrast, the presence of hyaluronan in direct contact with cultured cells overexpressing the CD44 receptor did not make a difference in the drug activity on cell viability. In the applied experimental conditions, cisPt uptake by the cells could not be evaluated. In theory, if drug alone and as complex were equally cytotoxic, the same amount of drug had supposedly entered the cell and reached the nuclear DNA. The presence of CD44 transmembrane receptor on the cell surface is claimed by many as a targeting strategy to increase the specificity of anticancer drugs [11]. The “targeting” concept cannot be demonstrated in vitro, as the drug delivery system is put in direct contact with its target cells. As for the possibility that hyaluronan-CD44 interaction favors drug uptake into the cell, the polymer is not internalized after binding to CD44. It activates intracellular signal transduction pathways responsible for the various activities of hyaluronan on cells [16]. Thus, NaHA should not lead to more cisPt entering the cell. Finally, Kim et al. recently proposed that hyaluronan interacts with CD44 involving only a limited number of receptors and that this number is independent of its molecular weight [26]. Thus, using LMW or HMW should have made no difference at the cellular level.

It is important to note that the cell viability in these experiments was measured at the same cisPt nominal concentration, whether applied alone or as a complex. In the two cases, cisPt concentration was the same; however, if the complex is present, only a fraction of cisPt from the complex would be free to enter the cell at a given time. The complex as such should not diffuse, in particular if hyaluronan is bound to CD44 and has high molecular weight. From this perspective, the complex may be considered more active than the drug alone because it caused the same cytotoxicity despite the complexed drug not being immediately available. Similar results of equal cytotoxicity with free and complexed cisPt were also observed by Zhang and co-workers. They formed a complex between cisPt and chondroitin sulphate A, a glycosaminoglycan very similar to NaHA in structure. The two polymers differ for the presence of sulphate groups that are absent in NaHA. They proved the existence of the complex and its effectiveness equal to cisPt alone in SW4800 human colon cancer cells and in HeLa human cervix cancer cells [27]. However, we also demonstrated that cisPt dissociates from the complex at a certain rate and, normally, release in NaCl 0.9% is complete in 48 h [10]. Assuming that the release from the complex occurred in the same way in the culture medium, the cytotoxicity measured at 24 h and 48 h would be determined by the released drug, i.e., with no difference compared to the treatment with the drug alone. Clearly, these last considerations bring us back to the second point of whether the complex was really present in the diluted solutions added to wells.

### 2.3. cisPt/NaHA Complex (Solution and Film) Slowed Down Spheroid Growth

In comparison with 2D cell monolayers, 3D cell culture models, known as spheroids, imitate tissue-like or organ-like characteristics that better replicate the cellular environment in vivo than what 2D cell cultures do. They more accurately mimic features of solid tumors such as spatial architecture, physiological responses, gene expression patterns, secretion of soluble mediators, and drug resistance mechanisms [28]. As per our knowledge, ours was the first experiment that evaluated the cytotoxicity of cisplatin/hyaluronan complex in spheroids using high molecular weight sodium hyaluronate. Spheroids were grown from VEM-responsive and VEM-resistant A375 cells, since they were most sensitive to the various cisPt-based treatments.

#### 2.3.1. Cytotoxic Activity Testing in VEM-Sensitive and Resistant A375 Spheroids

As observed with 2D cell models, the lowest cisplatin IC_50_ was achieved in VEM-sensitive A375 melanoma cells (Table 1). Thus, we grew 3D multicellular spheroids of melanoma using both responsive and resistant cells to vemurafenib.

In the first experiment, cisPt concentration was 1 µM for all treatment groups, namely:

- cisplatin alone;
- cisPt complexed with high molecular weight hyaluronan;
- cisPt combined with valproic acid;
- valproic acid alone;
- cisPt/NaHA HMW complex combined with valproic acid.

Thus, the drug concentration used (1 µM) was about half the IC_50_ determined in VEM-responsive A375 cells. Conversely, NaHA and valproic acid concentrations were 3.6 µM (to keep constant the 3.6:1 monomer:drug molar ratio) and 100 µM, respectively.

**Figure 4:**
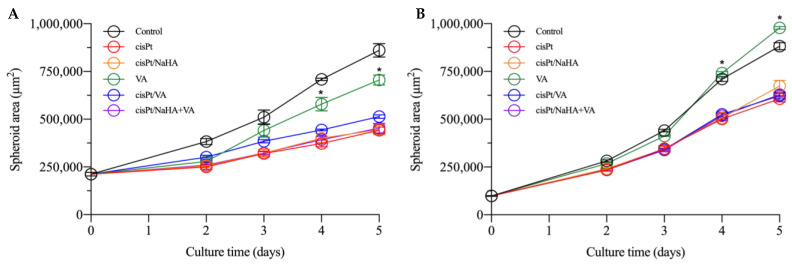
Area of VEM-responsive (**A**) and VEM-resistant (**B**) A375 spheroids as a function of culture time with various treatment groups: untreated control (black), cisPt alone (red), cisPt/NaHA HMW complex (orange), valproic acid (VA, green), cisPt + VA (blue), and cisPt/NaHA HMW + VA (purple). Data are reported as mean ± SEM (*n* ≥ 3). Asterisks indicate a statistically significant difference (*p* < 0.05).

Figure 4A shows that, for VEM-responsive spheroids, the cisPt/NaHA complex treatment was equally effective as cisPt alone. Similarly, the combination of the complex with valproic acid was also equally effective. Moreover, none of the treatments were effective in preventing spheroid growth, since the spheroid area increased compared to day 0 in all cases. However, in all groups the growth was significantly slowed down compared to the untreated control (*p* < 0.05 compared to the control and VA groups). The combination of cisPt with valproic acid again did not give the supposed synergism, which may have depended on the cell type [24].

Similar findings were also observed with VEM-resistant spheroids, with the same effect by all treatments (Figure 4B). Valproic acid alone was ineffective in both spheroid types.

**Figure 5:**
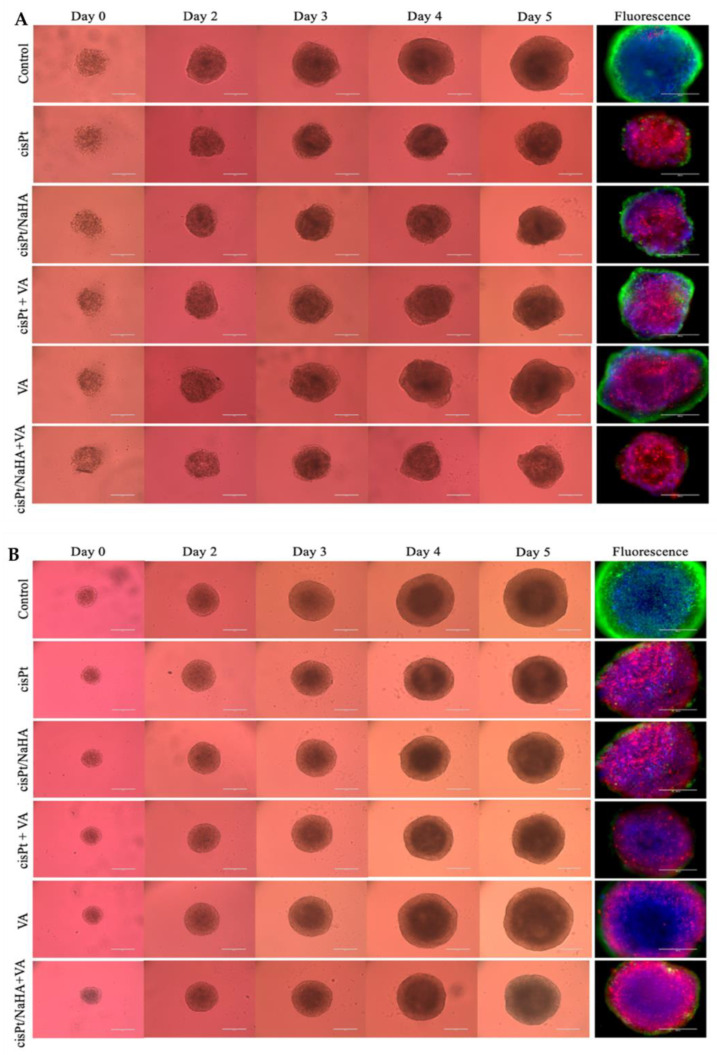
Representative images of (**A**) VEM-responsive A375 spheroids and (**B**) VEM-resistant A375 spheroids treated with no drug (control), cisPt alone, cisPt/NaHA HMW complex, cisPt + VA, VA alone and cisPt/NaHA HMW + VA at days 0–5 of treatment. The cisPt concentration was 1 µM in all cases, while the VA concentration was 100 µM. The last to the right series of fluorescence images depict cellular death of the spheroids in the various treatment groups. They are composite images of 4′,6-diamidino-2-phenylindole (DAPI) (blue), calcein acetoxymethyl (calcein AM) (green) and ethidium homodimer-1 (EthD-1) (red). Scale bar: 400 μm.

At the end of experiments, spheroids were stained. The blue, green, and red colors in Figure 5 identify the nuclei, live, and dead cells, respectively. The microscopic observation of stained VEM-responsive spheroids showed the total cell viability in control spheroids (green spots) (Figure 5). Conversely, in cisPt, cisPt/NaHA HMW, and cisPt/NaHA HMW + valproic acid, the numerous red spots confirmed the prevalence of dead cells as a result of significant cytotoxic action by cisPt, compared to the 2 VA-groups (with or without cisplatin). The same results were achieved in VEM-resistant spheroids, where the fluorescent images confirmed that the combination of cisPt and VA was neutral. Indeed, based on the very few visible live cells (the green spots), the combination of cisPt/NaHA complex with valproic acid seemed to work better. As an additional observation, the surface of the spheroids treated with cisPt, cisPt/NaHA complex or the complex with VA exhibited rough morphology, whereas the control spheroids and the VA-treated ones were smooth and uniform. This different morphology is related to the extent of the cytotoxic effect.

**Figure 6:**
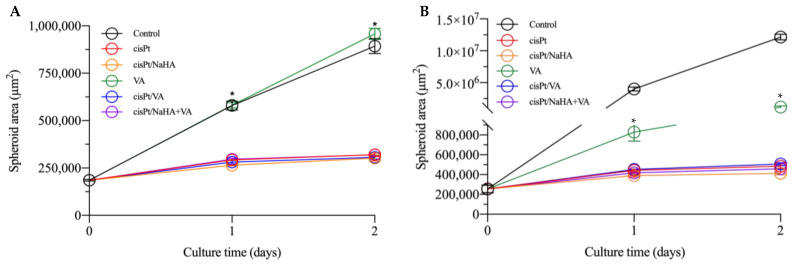
Area of VEM-responsive (**A**) and VEM-resistant (**B**) A375 spheroids as a function of time with various treatment groups: untreated control (black), cisPt alone (red), cisPt/NaHA HMW complex (orange), valproic acid (VA, green), cisPt + VA (blue), and cisPt/NaHA HMW + VA (purple). Data are reported as mean ± SEM (*n* ≥ 3). Asterisks indicate a statistically significant difference (*p* < 0.05).

In the second experiment with spheroids, the increase of cisPt concentration from 1 to 2.5 µM (very close to the IC_50_) led to similar results in both VEM-responsive and VEM-resistant spheroids. NaHA concentration was kept at about 9 µM to have the same molar ratio between monomer and drug (3.6:1), while the VA concentration was kept equal to that in the previous experiment (100 µM). As shown in Figure 6, the treatments with cisPt/NaHA complex and cisPt alone in VEM-responsive A375 spheroids were equivalent in terms of spheroid area (Figure 6A). Neither the treatment with VA, alone or in combination with cisPt or the cisPt/NaHA complex, improved the anticancer activity of cisplatin in this melanoma cell line. VA-treated spheroids exhibited behavior similar to the control.

**Figure 7:**
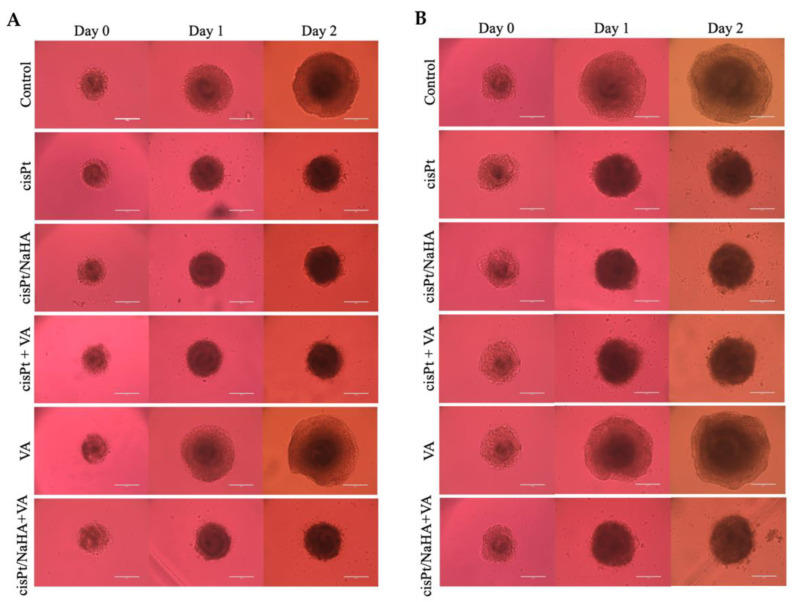
Representative images of (**A**) VEM-responsive A375 spheroids and (**B**) VEM-resistant A375 spheroids treated with no drug (control), cisPt alone, cisPt/NaHA HMW complex, cisPt + VA, VA alone and cisPt/NaHA HMW + VA at days 0–2 of treatment. The cisPt concentration was 2.5 µM in all cases, while the NaHA and VA concentrations were 9 and 100 µM, respectively. Scale bar: 400 µm.

Notably, in both experiments, there was a clear difference in the initial spheroid area at day 0 and the extent of growth between VEM-responsive and VEM-resistant A375 spheroids as a function of time up to 2 days. It reflected the higher aggressiveness of the latter cell line, a consequence of the acquired resistance to vemurafenib. Indeed, during the second experiment, the observation had been interrupted after only 2 days of treatment due to the uncontrolled growth of control spheroids (Figure 7). In fact, if the area of control spheroids had fallen outside the range measurable by optical microscopy, we would have lacked the control value for comparison with the treated spheroids.

The fact that the resistant cells reached confluence more rapidly and formed bigger spheroids at time 0 as compared to VEM-sensitive cells is in line with reports in the literature. In a 3D spheroid model, Sandri et al. found that the invasion index of vemurafenib-resistant melanoma cells was greater than that in the non-resistant counterpart [29].

With the exception of the spheroids treated with VA alone, those treated with cisPt in the various combinations always had an area lower than the control at all time points. Valproic acid alone was ineffective as it is not an anticancer drug. The cell type did not make a difference in the effect of various treatments. In VEM-resistant A375 cells, VA even let the spheroids become as big as the control (Figure 7B). All these findings supported the idea that, in melanoma spheroids, the cisPt/NaHA complex attenuated tumor growth by the same mechanism as cisPt alone did.

As previously reported, the 3D cell models advance in the anticancer drug studies in vitro by better replicating the in vivo cellular environment compared to the 2D cells. Still, spheroids are not a full organism where the drug is absorbed, distributed, metabolized, and excreted. Thus, it is unlikely that the lower nephrotoxicity of cisPt when complexed with NaHA, which has been previously demonstrated in vivo [9], can be confirmed in a cell model. In the present work, nephrotoxicity was actually assessed in a kidney cell line (HEK293) by comparing the toxicity of cisPt alone with that of cisPt/NaHA HMW and cisPt/NaHA LMW complexes. Cell viability was reduced in a concentration-dependent manner similarly bythe three treatments (Table S1).

#### 2.3.2. cisPt/NaHA Film Inhibited the Development of Spheroid-Derived New Cell Colonies

**Figure 8:**
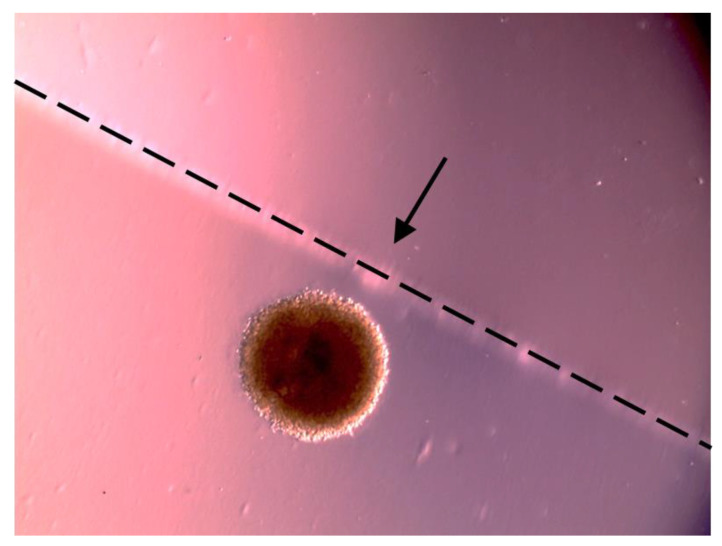
Optical microscopy image of the spheroid leaning on the film. The dashed line and the arrow indicate the outer edge of the film.

Due to its 3D structure and size, the spheroid model allowed the testing of the cytotoxic effect of the whole film, providing experimental conditions suitable to mimic the loco-regional application of the film and prolonged release of cisPt from the system containing the complex. Different from the previous two experiments with drug solutions on spheroids, here it was necessary to grow larger and more resistant spheroids that could withstand being transferred, when ready, to the larger well hosting the film (or the reference drug solution). The film was laid flat at the bottom of the well when the VEM-resistant A375 spheroid was gently deposited on top of it (Figure 8). The film was cut to a size that resulted in a cisPt concentration of 100 µM in the well. Conversely, for the reference treatment a solution of 25 µM cisPt alone was used. This solution was more concentrated than in the previous experiments, because spheroids were bigger when the treatment started. The concentration was also 10 times higher than the IC_50_ measured in VEM-responsive A375 melanoma cells. However, according to the experimental protocol, such a high concentration was progressively diluted to zero in the first 6 h of the experiment, to mimic the in vivo clearance of the cisPt solution from the application site. This was not done with the film, which is supposed to remain longer on site and release the drug continuously. The difference in initial drug concentration between film treatment and reference treatment was also deliberate, considering that cisPt would be 100% available from the solution, while the hyaluronan film would release it in a slow prolonged manner.

**Figure 9:**
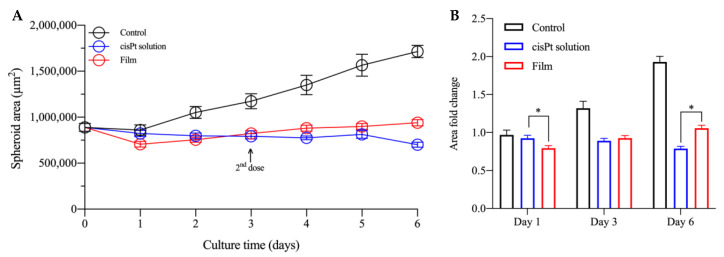
(**A**) Area of VEM-resistant spheroids as a function of culture time with various treatment groups: untreated control (black), cisPt solution (blue) and film (red). (**B**) Fold change in the area of VEM-resistant A375 spheroids treated during culture time vs. Day 0. Data are reported as mean ± SEM (*n* ≥ 6). Asterisks indicate a statistically significant difference (*p* < 0.05).

The first result obtained in this experiment showed that both treatments inhibited spheroid growth as compared to the control (Figure 9). In fact, without treatment, the spheroids doubled their area in 6 days, whereas the area remained substantially unchanged under the effect of the drug. The treatment with the cisPt solution was very effective, considering that the drug was added at very high concentration, but also washed away within 6 h. The spheroid area remained rather constant and, when the solution was dosed again at Day 3, it likely found dead cells with a negligible effect on the spheroid area.

**Figure 10:**
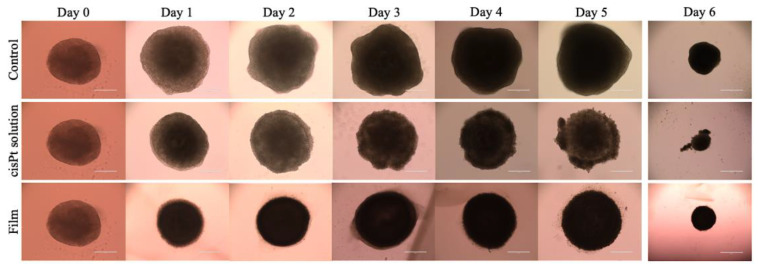
Representative images of VEM-resistant A375 spheroids treated with cisPt solution (25 µM) and film (cisPt 100 µM) following 6 days of treatment. Until Day 5 the magnification was 10× (scale bar: 400 µm), then it was 4x on the last day (scale bar: 1000 µm).

In the first day of treatment with the film, there was a certain reduction in the area of spheroids compared to the drug solution (*p* < 0.05), but the area increased again in the next days, slightly and continuously up to Day 6, thus the meaningfulness of such initial reduction is uncertain. One difference of the spheroids treated with the film was their morphology that was more spherical with a regular outline as compared to the other two groups (Figure 10).

**Figure 11:**
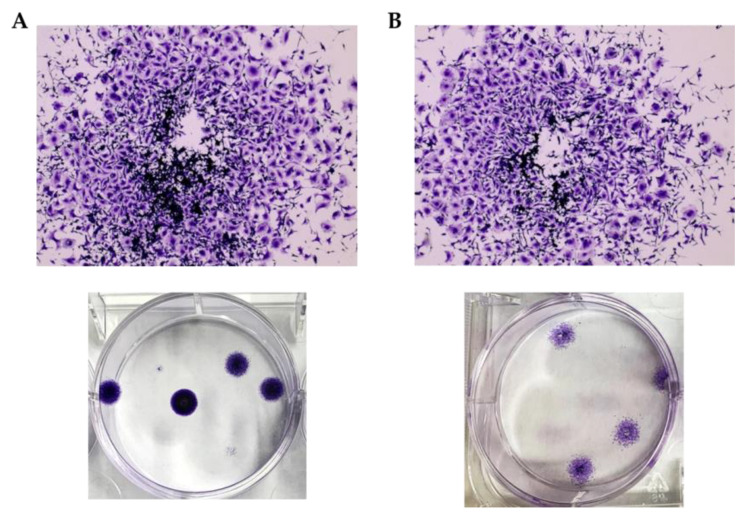
Crystal violet-stained images for control (**A**) and cisPt solution (**B**) groups.

One novel and interesting result of this experiment was that new cell colonies were formed during the experiment time only in the group treated with the cisPt solution. Indeed, to apply the second dose, the spheroids were transferred to a new 6-well plate and crystal violet assay was performed on the old one. The assay evidenced the presence of cell colonies in untreated and cisPt solution-treated groups. Conversely, no colonies were formed in the group treated with the film. This result was impressive because, during the culture time, some cells can detach from the spheroid and grow on the well surface. The usual plates for spheroid culture have an ultra-low attachment surface to avoid spheroid attachment to the bottom of the well, which would prevent the formation of the 3D characteristic structure. However, in this experiment we used 6-well plates whose bottom was coated with materials that promoted the growth of cells in the monolayer (2D cell models). Thus, the cells that were detached from the spheroids could adhere to the bottom of the well and grow in a monolayer, giving rise to new cellular colonies. In the spheroids treated with film, no cells were observed attached to the bottom of the well (Figure 11).

Thus, the real noteworthy effect of the cisPt/NaHA complex was the inhibition of colony formation. Likely, this was possible because the film remained in contact with the cells and kept releasing the drug daily. Conversely, the drug applied as solution was washed away in 6 h. This action, translated to the in vivo situation, suggests that the film could stop cells originating from the primary tumor from developing local metastases and causing tumor relapses. This appears to be a relevant advantage of the film by considering how often the primary tumor can acquire metastatic potential due to cells surviving surgery/chemotherapy, which could spread over to other body sites. The crystal violet assay was also repeated at the end of the experiment (Day 6). Once again, the assay showed the presence of new colonies only in the control and cisPt solution-treated groups.

## 3. Materials and Methods

### 3.1. Materials

cis-Diammineplatinum(II) dichloride (cisplatin, cisPt) and valproic acid (VA) were purchased from AK Scientific Inc. (Union City, CA, USA). High molecular weight (HMW) hyaluronic acid (1330 kDa; HA ophthalmic, batch A19270), which was obtained as the sodium salt (NaHA), was kindly donated by Fidia Farmaceutici S.p.A. (Abano Terme, PD, Italy). Low molecular weight (LMW) hyaluronic acid (10 kDa), also obtained as the sodium salt, was acquired from Creative PEGWorks (Durham, NC, USA). Since NaHA exists as the polymer with different molecular weights, it is also referred as the polymer in certain places in the paper. MTT (3-(4,5-dimethylthiazol-2-yl)-2,5-diphenyltetrazolium bromide) and phosphate-buffered saline (PBS) were supplied by Fisher Scientific (Hampton, NH, USA). Dulbecco’s modified Eagle’s medium (DMEM), Roswell Park Memorial Institute (RPMI) 1640 media and cell culture grade water were purchased from ThermoFisher Scientific Inc. (Waltham, MA, USA), whereas Fetal Bovine Serum (FBS) was from Atlanta Biologicals (Oakwood, GA, USA).

### 3.2. Complexation Efficiency between Cisplatin and Hyaluronan

We determined the complexation efficiency, i.e., how much polymer was needed to coordinate all cisPt available in solution, by considering the monomer to drug molar ratio and polymer molecular weight as variables. Here, the term “monomer” refers to the repeating unit of a dimer in NaHA formed by N-acetyl-D-glucosamine and D-glucuronic acid linked by a ß-1,3-glycosidic bond (MW: 379.32 Da). Two hyaluronans with molecular weights of 10 kDa and 1330 kDa were used. An aqueous solution of cisPt was freshly prepared at the final concentration of 0.066% (*w/v*) (0.66 mg/mL; 2.2 mM), which is also the concentration of cisPt in the film-forming mixture (FFM). Then, 4 mL of this cisPt solution was transferred to vials containing accurately weighed amounts of NaHA powder (4–48 mg). Consequently, NaHA concentration varied in the range 1–12 mg/mL (2.6–31.6 mM). The resulting molar ratios ranged from 1.2:1 to 14.4:1 (monomer:drug). These solutions were stirred with magnetic bars for 18 h to allow for the polymer hydration. The maximum NaHA concentration tested was 12 mg/mL, because at higher concentration the solution was too viscous. Finally, a sample from each solution was collected, appropriately diluted, and injected in HPLC to quantify free cisPt at 18 h by the RP-HPLC method described in Section 3.3.

### 3.3. HPLC Analysis

The quantification of cisplatin and NaHA, when needed, was carried out on a Waters HPLC system (Waters Corporation, Milford, MA, USA) equipped with e2695 Separations module and UV-visible detector set at 210 nm. The column was a Synergi Polar-RP (4 µm, 4.6 × 150 mm; Phenomenex, Torrance, CA, USA). Isocratic elution at room temperature was performed with 25 mM KH_2_PO_4_, adjusted to pH 5.8 ± 0.1 with 1 M KOH at a flow rate of 0.6 mL/min. The injection volume was 10 µL. Empower 3 Feature Release 3 software was used to analyze the output signal. In these conditions, the retention times of NaHA and cisPt were 1.5 and 3.4 min, respectively. The method linearity was confirmed at 1–100 µg/mL range (y = 13.876x − 0.3633; R^2^ = 1.0000) for cisplatin and at 20–250 µg/mL range for sodium hyaluronate (y = 1.8661 + 2.0037; R^2^ = 0.9999).

### 3.4. Experiments with 2D Cell Models

#### 3.4.1. Preparation of cisPt and cisPt/NaHA Stock Solutions

All test solutions for the studies in 2D cell models were prepared from a cisPt stock solution in cell culture grade water (Fisher Scientific, Hampton, NH, USA), which was then diluted at the final concentration of 0.066% (*w/v*) (2.2 mM). As said, this was the cisPt concentration in the film-forming mixture (FFM).

The cisPt/NaHA complex stock solution was prepared by adding an accurately weighed amount of solid polymer (HMW NaHA) to a given volume of cisPt stock solution to a final NaHA concentration of 0.3% (*w/v*) (7.9 mM as monomer). An approximately 8–9 times lower concentration of NaHA than that in the FFM (65.9 mM as monomer) was used to maintain a low viscosity. The system was stirred for 18 h until the polymer dissolved completely. An identical procedure and the same NaHA concentration were adopted to prepare the cisPt/NaHA complex solution with LMW hyaluronan.

In the obtained cisPt/NaHA solutions the molar ratio between hyaluronan and drug was 3.6:1, irrespective of the polymer MW, because the moles were calculated based on the MW of the repeating unit (monomer MW: 379.3 Da). As a note, the original formulation of the film (see Section 3.5) contained a monomer to drug molar ratio of 30:1. The formation of cisPt/NaHA complex with both hyaluronan polymers was assessed by RP-HPLC (see Section 3.3).

The stock solution with cisPt + valproic acid was prepared by adding the VA solution in dimethyl sulfoxide (DMSO) to the cisPt stock solution to a final VA concentration of 400 µM.

Prior to the addition to wells, each drug solution (cisPt alone, cisPt/NaHA, cisPt + VA) was serially diluted with cell culture medium (Table 2).

#### 3.4.2. Cell Culture

MIA PaCa-2 and BxPC_3_ human pancreatic cancer cells, A375 human melanoma cells, and A549 human lung carcinoma cells were purchased from American Type Culture Collection (Manassas, VA, USA). MIA PaCa-2, BxPC_3_ and A375 cells were grown in DMEM medium, whereas A549 cells were maintained in RPMI medium. Media were supplemented with 10% FBS and penicillin-streptomycin mixture at 37 °C and 5% CO_2_ with 95% relative humidity. A549 cells required additional supplementation of 1% sodium pyruvate (VWR scientific, PA, USA).

The primary A375 cell line responds to the treatment with the anticancer drug vemurafenib (VEM) A VEM-resistant A375 cell line was also generated according to Rathod [30] to test the complex on a more aggressive cancer cell line. To do so, A375 primary cells were treated with the 0.2 µM VEM up to 20 passages. When resistance was achieved, the drug was withdrawn, and the resistant cell line was maintained in the usual medium (DMEM) for the experiments. Rathod et al. [30] proved the acquired resistance by comparing the cytotoxicity of vemurafenib in parental and newly developed resistant-cell lines.

#### 3.4.3. In Vitro Cytotoxicity Experiments

The cytotoxic activity of cisplatin complexed with LMW or HMW hyaluronan or in combination with valproic acid was evaluated on the selected cell lines using 3-(4,5-dimethylthiazol-2-yl)-2,5-diphenyl tetrazolium bromide (MTT) assay.

Two different experiments were performed. According to the first protocol, cells were seeded in 96-well plates at the density of 5000 cells per well and incubated overnight. The stock drug solutions prepared (see Section 3.4.1) were serially diluted with cell culture medium to the final concentration ranges of cisPt, NaHA, and VA reported in Table 2. The hyaluronan used was the HMW only. Following 48 h of incubation with the drug solution, the cell viability was measured by MMT colorimetric assay. MMT dye was dissolved in PBS pH 7.4 at 5 mg/mL concentration, and then 10 µL of this solution was added to each well and cells were incubated for 3 h (37 °C, 5% CO_2_). Afterwards, the reagent was washed away, and the formed MMT-formazan crystals were dissolved by adding 100 µL of DMSO to each well. Plates were shaken well before UV-spectrometric analysis at 570 nm wavelength (BioTek Instruments, Inc., Winooski, VT, USA).

For the second experiment, cells were seeded and treated in the same way. Either LMW or HMW NaHA was used. The drug solutions of Section 3.4.1, with or without NaHA, were properly diluted to have a cisPt concentration of either 2 or 10 µM in the medium for cell treatment. The dilution did not change the monomer to drug molar ratio, which was fixed at 7:1. The effect of drug treatment was assessed after 4 and 24 h by MTT assay, as described above.

### 3.5. Film-Forming Mixture Preparation and Film Manufacturing

The viscous solution of the film-forming mixture (FFM) was prepared according to the original polymeric film composition [10]. Briefly, plasticizing agents, i.e., PEG 200 and sorbitol (as solution 70% *w/v*) plus PEG 1000 stearate, were dissolved in purified water. Then, the film-forming polymer PVA was added to the solution, and the mixture was heated at 75 °C for 2 h to facilitate the dissolution of PVA in the solution. After cooling the solution back to 25 °C, cisplatin was added under stirring. After complete drug dissolution, hyaluronan was added as the last component because its complete hydration, which occurred after 18 h of mechanical stirring, made the film-forming mixture extremely viscous. The composition by weight of films is presented in Table 3.

The films were manufactured by pouring the film-forming mixture directly onto a Petri dish (diameter 5–8 cm), achieving a thickness of 2 mm. They were allowed to air dry at T ≤ 25 °C for two days. This manufacturing method had the advantage of gravimetrically controlling the amount of film-forming mixture used [10,31]. The drying phase ended when the weight of the films remained constant.

### 3.6. Formation and Treatment of 3D Multicellular Tumor Spheroids

#### 3.6.1. Treatment with Drug Solutions

A375 melanoma cells, either responsive or resistant to vemurafenib (VEM), were seeded at a density of 1500 cells/well in an ultra-low attachment 96-well plate for spheroids (Corning Life Sciences, Corning, NY, USA). Spheroid microplates were sealed with tape and centrifuged at 130× *g* for 10 min (4 °C) prior to culture in the incubator at 37 °C, and 5% CO_2_ for 72 h to form melanoma tumor spheroids resistant enough for the subsequent manipulations. After the spheroids were developed, the treatment could start. The spheroids were divided into 6 groups, namely, control group (untreated); cisPt; cisPt/NaHA HMW; VA; cisPt + VA; and cisPt/NaHA HMW + VA. Concentrations of cisPt, NaHA, and valproic acid are given in Table 4. The untreated control received the plain medium.

At time 0, the medium was removed from the well and 200 µL of test solution was added. Then, every other day, half of the medium was replaced with an equivalent volume of fresh medium containing cisPt or cisPt + VA at twice the concentration to test, until the end of the experiment. By doing so, the cells were maintained under the effect of constant drug concentration throughout the whole experiment duration. On a daily basis, the spheroid growth was visually monitored using an Evos imaging system (ThermoFisher Scientific, Waltham, MA, USA). In particular, the surface area was recorded as an indicator of the mass growth. The spheroid growth was expressed as daily fold change area relative to time 0 when the treatment was initiated. The duration of the experiment varied from 2 to 5 days depending on how much the spheroids grew under the effect of each treatment. The faster they grew, the shorter the experimental time. On the last day of the experiment, spheroids were stained with a mixture of three dyes: 1 µM calcein acetoxymethyl (calcein AM) for the green color, 6 µM ethidium homodimer-1 (EthD-1) for the red color and 4 μg/mL 4′,6-diamidino-2-phenylindole (DAPI) for the blue color (Santa Cruz Biotechnology, Dallas, TX, USA). The dyes were dissolved in sterile phosphate-buffered saline (PBS) and the obtained solution was used to completely replace the drug-containing medium in the well. Spheroids were then incubated with the dye solution for 3 h to let the dye penetrate inside the spheroid before imaging. Finally, fluorescent images were taken using an Evos fluorescence microscope (ThermoFisher Scientific, Waltham, MA, USA). The color of live and dead cells and nuclei were green, red, and blue, respectively.

#### 3.6.2. Treatment of Spheroids with the Film

The film used in the experiments with spheroids was manufactured as described in Section 3.5. The preparation of spheroids was as described above with one difference. To test the hyaluronan film directly on spheroids, it was necessary to grow the cells to an initial area of 1 million µm^2^ prior to treatment, instead of the 20,000–30,000 µm^2^ that was suitable for the previous experiment. The larger the area of spheroids, the greater their integrity and resistance, which was required to transfer them safely from the 96 well-plate to a 6 well-plate. The larger well (9.6 cm^2^ vs. 0.32 cm^2^ for the 96 well-plate) allowed to hold the film flat at the bottom of the well. A piece of film was cut and weighed. The size of the piece was such that the cisPt concentration in contact with the spheroid would be 100 µM. The reference treatment group received a 25 µM cisPt solution in cell culture grade water added to the well at time 0. Then, at every 30 min for 6 h, half of the volume of the solution in the cisPt solution group was removed from the well and replaced with fresh medium without the drug. This caused a progressive dilution to zero of the drug in the well. This protocol was adopted to mimic the cisplatin clearance from the site of application, which is expected to be faster for the solution than for the film. The 30-min frequency was decided considering that cisPt shows a monoexponential decay in plasma with a half-life of 20 to 30 min following the intravenous administration of 50 or 100 mg/m^2^ [32].

As before, the spheroid growth was monitored and the daily fold change in the area relative to time 0 was calculated. Time 0 was when treatment started with the “first dose”. After 72 h (end of Day 3), the spheroids were transferred to a new 6 well-plate for the “second dose”. For each treatment, the same volume of cisPt solution or a new film were applied. The cisPt solution was progressively diluted as done for the first dose. The spheroids were monitored up to the end of Day 6 from the beginning of the experiment. After taking the last picture, the spheroids were stained, and the crystal violet colorimetric assay was performed to visualize the presence of newly developed cell colonies. Crystal violet assay was also performed on the well left empty after spheroid transfer for 72 h. For crystal violet assay, the 6-well plates were washed with PBS and glutaraldehyde (10% *v*/*v*) was added, followed by incubation for 20 min. Then, plates were washed again and incubated for 30 min with crystal violet (5 mg/mL). Finally, the plate was washed and dried at room temperature. Plates were observed and the colony quantification was carried out by the Evos imaging system (ThermoFisher Scientific, Waltham, MA, USA).

### 3.7. Statistical Analysis

Cytotoxicity data from a minimum of three replicates are reported as the mean ± standard error of the mean (SEM). Differences were evaluated for statistical significance by unpaired two-tailed Student’s *t*-test using GraphPad Prism (GraphPad 8.0.1 Software, La Jolla, CA, USA). A *p*-value less than 0.05 was considered to indicate statistical significance.

## 4. Conclusions

This study showed that there were no statistically significant differences in the cytotoxicity of cisplatin and cisPt/NaHA complex in 2D cancer cell cultures, and there was no difference even when cisPt was combined with valproic acid. These results demonstrated that the anticancer activity of cisplatin was not compromised by complexation with hyaluronic acid. Irrespective of CD44 receptors, the cellular uptake in cell monolayer could be similar for drug and cisPt/NaHA complex. Therefore, in the 2D cell model, the CD44-hyaluronic acid targeting mechanism may not be conclusive, because the delivery system is directly placed in contact with the target cells. Even in multicellular 3D spheroid, the cisplatin and cisPt/NaHA complex showed comparable activity in terms of spheroid growth. It is worth recalling that the first in vivo experiments with the film had indicated that the superiority of the complex in the film was rather linked to different pharmacokinetics than cisplatin applied as an intrapleural solution. The complex slowed the absorption of cisPt into blood circulation, which prolonged its loco-regional action. Moreover, it gave rise to high plasma levels of platinum, but at the same time modified and, indeed, decreased the distribution of the drug toward organs susceptible to cisplatin toxicity. In light of this, spheroids worked better than monolayer cells in simulating the “kinetic aspect”, because the spheroid could be placed in direct contact with the film, mimicking the loco-regional application. By doing so, noteworthily, the inhibition of the growth of new cell colonies resulting from the spheroid itself was assessed. These findings recommend further investigation of the effectiveness of cisPt/NaHA complex in an animal model of melanoma.

## Figures and Tables

**Table 1:** IC_50_ of cisPt, cisPt/NaHA HMW and cisPt + VA in BxPC_3_, A549, MIA PaCa-2, and VEM-responsive A375 cells. Data are reported as mean ± SEM (*n* = 6).

Cell Line	IC_50_ (µM)		
	cisPt	cisPt/NaHA HMW	cisPt + VA
BxPC_3_	9.63 ± 0.05	10.58 ± 0.08	NA
A549	9.44 ± 0.10	9.14 ± 0.07	10.71 ± 0.05
MIA PaCa-2	5.04 ± 0.04	5.03 ± 0.11	5.11 ± 0.05
VEM-responsive A375	2.17 ± 0.01	2.32 ± 0.04	2.39 ± 0.06

**Table 2:** Final drug and NaHA concentrations for the first experiment.

Test Solution	cisPt (µM)	NaHA HMW (µM)	VA (µM)
1	0.2–25.0	0	0
2		0.7–180.5	0
3		0	0.4–100.0

**Table 3:** Hyaluronate film composition by dry weight (% *w*/*w*).

Component	% (*w*/*w*)
Sodium hyaluronate (NaHA)	40.7
PVA 83,400	14.7
PEG 200	14.7
PEG 1000s	14.7
Sorbitol solution (70% *w*/*v*)	14.7
Cisplatin (cisPt)	0.5

**Table 4:** Solution composition for the treatment of 3D spheroids.

Treatment Group	cisPt (µM)	NaHA (HMW) (µM)	VA (µM)
1	1 or 2.5	0	0
2	1 or 2.5	3.6 or 9.0	0
3	0	0	100
4	1 or 2.5	0	100
5	1 or 2.5	3.6 or 9.0	100

## Data Availability

Data supporting reported results can be found in our previous research paper [10].

## Supplementary Materials

The following supporting information can be downloaded at: https://www.mdpi.com/article/10.3390/ijms242115725/s1.
