# Supplementary material for: In Vitro Assessment of Cisplatin/Hyaluronan Complex for Loco-Regional Chemotherapy
Source: Int J Mol Sci. 2023 Oct 29;24(21):15725. doi: 10.3390/ijms242115725 (PMC10647681; doi:10.3390/ijms242115725)
Supplement: Supplementary file 1 [file ijms-24-15725-s001.zip › ijms-2603451-supplementary.pdf]

International Journal of
Molecular Sciences

MDPI

Article

In Vitro Assessment of Cisplatin/Hyaluronan Complex for Loco-Regional Chemotherapy

Supplementary Materials

Table S1. Percentage viability of HEK293 cells after 2 h exposure to cisPt alone, cisPt/NaHA HMW complex and cisPt/NaHA LMW complex as solutions. Three cisPt concentrations were tested, i.e., 2, 10 and 20 μM for all treatments. Data are reported as mean ± SEM (*n* ≥6).

Treatment Solutions	HEK293 cell viability (%)		
	**2 μM cisPt**	**10 μM cisPt**	**20 μM cisPt**
cisPt	73.16 ± 0.75	47.37 ± 1.31	38.93 ± 0.75
cisPt/NaHA HMW	61.01 ± 1.56	44.58 ± 1.02	35.78 ± 1.56
cisPt/NaHA LMW	59.88 ± 1.68	49.06 ± 0.90	34.16 ± 1.68

Materials and Methods

Cell culture: HEK293 human embryonic kidney cells were purchased from American Type Culture Collection (Manassas, VA, USA). Cells were grown in DMEM medium supplemented with 10% FBS and penicillin-streptomycin mixture at 37 °C and 5% CO_2 with 95% relative humidity.

Preparation of treatment solutions: All treatment solutions for the studies in 2D HEK293 cell model were prepared from a cisPt stock solution in cell culture grade water (Fisher Scientific, Hampton, NH, USA) as reported in Section 3.4.1. of the main text. The cisPt/NaHA complex stock solution with either molecular weight, was prepared by adding an accurately weighed amount of polymer powder to a given volume of the cisPt stock solution to a final NaHA concentration of 0.3% (*w*/*v*). The system was stirred for 18 h to dissolve the polymer completely. Prior to the addition to wells, each stock solution (cisPt alone, cisPt/NaHA HMW, and cisPt/NaHA LMW) was diluted with cell culture medium to the desired test concentrations of cisPt, i.e., 2, 10 and 20 μM (Table S1).

In vitro cytotoxicity studies: HEK293 cells were exposed to each treatment for 2 hours. Following drug treatment, cell viability was by MTT assay as described in Section 3.4.3 of the main text.
